# HR-SEM and FT-IR dataset for green corrosion inhibition activity of 4-{[4-(pyridin-2-yl)piperazin-1-yl]methyl}aniline at CO_2_ atmosphere

**DOI:** 10.1016/j.dib.2021.107492

**Published:** 2021-10-18

**Authors:** Raman Govindhan, Srinivasan Anbalagan, Meenakshisundaram Ravishankar

**Affiliations:** a Department of Chemistry, Sri Moogambigai Arts & Science College (women), Affiliated to Periyar University, Dharmapuri, Tamilnadu 636 805, India; b Department of Chemistry, Laxminarayana College of Arts & Science (women), affiliated to University, Dharmapuri, Tamilnadu 636 705, India; c Department of Chemistry, Rajah Serfoji Government College (Autonomus), Thanjavur, Tamilnadu 613 005, India

**Keywords:** PMMA, Novel catalyst, Corrosion inhibition, HR-SEM, NMR, FT-IR data

## Abstract

Mild steel (MS) corrosion inhibiting aptitude of 4-{[4-(pyridin-2-yl)piperazin-1-yl]methyl}aniline (PPMA), is presented [1], [2], [3] in this data set. Explorations of synthesized PPMA are carried out by FT-IR spectral analysis, nuclear magnetic resonance (NMR) and high resolution scanning electron microscopy (HR-SEM). The inhibition activity is investigated by potentio-dynamic method. The FT-IR and NMR results revealed that structure. These data sets are ideal tool for the applications like physical and chemical-engineering field.

## Graphical abstract

**Figure:**
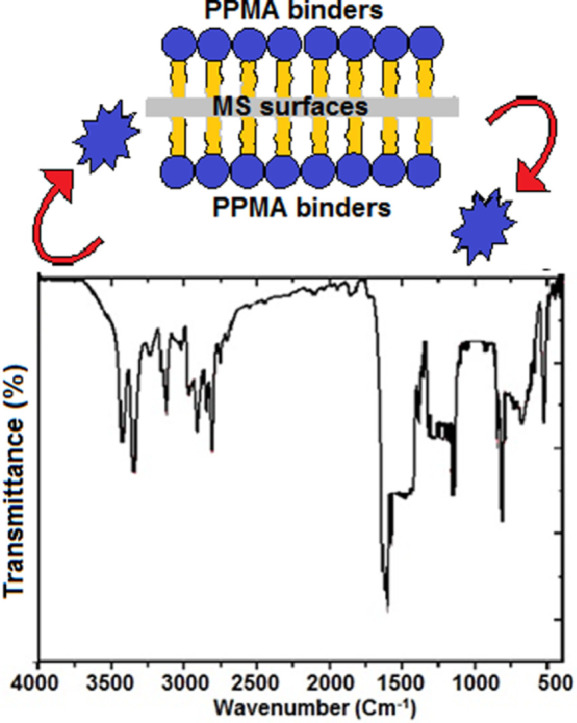

## Specifications Table

**Table:** 

Subject	Chemical Science
Specific subject area	Physical chemistry and Chemical engineering
Type of data	Table, Figure and Schemes.
How data was acquired	Spectroscopic and microscopic data used to the (corrosion resistive) engineering applications is explored.
Data format	Raw data.
Parameters for data collection	NMR and FT-IR spectra were recorded, FT-IR spectra were recorded with thermo scientific spectrometer, model no. iS5 equipped with attenuated total reflectance (ATR) competence which is implemented by Zn-Se crystal detector. Each spectrum was recorded with an achievement time of 18 s. The FT-IR dimension was scanned at a range from 4000 to 400 cm^−1^.
Description of data collection	FT-IR and NMR spectral consequence reveals that structure and purity of the catalyst such as PPMA [4]. HR-SEM morphology of as-synthesized PPMA and MS interacted PPMA is predicted.
Data source location	Department of Chemistry, Laxminarayana College of Arts & Science, Dharmapuri, Tamilnadu, India, and SAIF-IIT Madras.
Data accessibility	The data is available with this article

## Value of the Data

• The data were represent a valuable collection of the intact individual spectroscopic and microscopic data of PPMA interacted MS.
• The microscopic images and plots provide a novel way to look at the effectiveness of the corrosion inhibition potential of PPMA and further evolutions for other researchers to expand the future outcomes [2], [3], [4], [5].
• The FT-IR data provides specifying binding frequencies of the PPMA with MS [6].
• This data allows other researchers to explore or extend the corrosion inhibition activity analysis of petroleum oil storage containers at different atmosphere.

## 1 Data Description

**Fig. 1:**
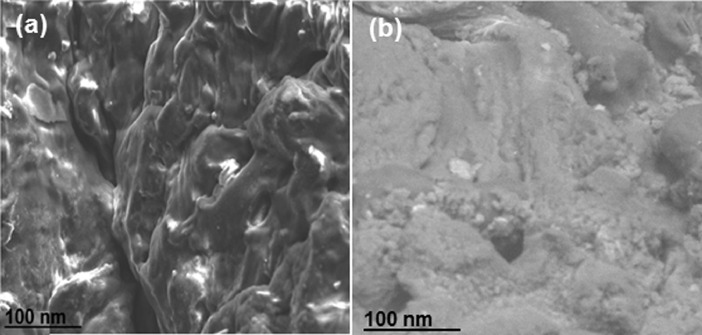
HR-SEM morphologies of (a) Bare PPMA and (b) PPMA interacted MS surfaces.

**Fig. 2:**
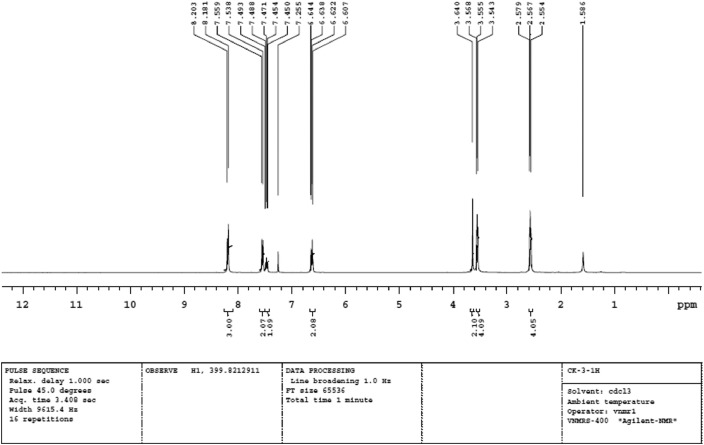
Proton NMR spectra of PPMA.

We present data includes the more information on (See Tables 1 and S1) the HR-SEM data of PPMA interaction with MS and Fig. 1 show the morphology of the reported materials. The Fig. 2 and Schemes S1 and 1 shows the synthetic scheme and corrosion inhibition efficiency data in (See Table 2). The resulted data are provided in Supplementary Table S1.

**Table 1:** MS corrosion inhibition data of PPMA at CO_2_ atmosphere.

Applied Potential (V)	Time (s)	WE (1).Current (A)	Index	WE (1).Potential (V)
−1.39755	6.64484	−0.00745	1	−1.39679
−1.39511	6.88884	−0.00744	2	−1.39465
−1.39267	7.13284	−0.00744	3	−1.39252
−1.39023	7.37684	−0.00743	4	−1.39069
−1.38779	7.62084	−0.00743	5	−1.38763
−1.38535	7.86484	−0.00743	6	−1.3858
−1.3829	8.10884	−0.00742	7	−1.38275
−1.38046	8.35284	−0.00742	8	−1.38062
−1.37802	8.59684	−0.00741	9	−1.37787
−1.37558	8.84084	−0.00741	10	−1.37573
−1.37314	9.08484	−0.0074	11	−1.37268
−1.3707	9.32884	−0.0074	12	−1.37085
−1.36826	9.57284	−0.00739	13	−1.36749
−1.36581	9.81684	−0.00739	14	−1.36597
−1.36337	10.0608	−0.00739	15	−1.36353
−1.36093	10.3048	−0.00738	16	−1.36047
−1.35849	10.5488	−0.00738	17	−1.35834
−1.35605	10.7928	−0.00737	18	−1.35559
−1.35361	11.0368	−0.00737	19	−1.35315
−1.35117	11.2808	−0.00736	20	−1.35071
−1.34872	11.5248	−0.00736	21	−1.34857
−1.34628	11.7688	−0.00735	22	−1.34644
−1.34384	12.0128	−0.00735	23	−1.34338
−1.3414	12.2568	−0.00735	24	−1.34125
−1.33896	12.5008	−0.00734	25	−1.3385
−1.33652	12.7448	−0.00734	26	−1.33636
−1.33408	12.9888	−0.00733	27	−1.33392
−1.33163	13.2328	−0.00733	28	−1.33148
−1.32919	13.4768	−0.00732	29	−1.32965
−1.32675	13.7208	−0.00732	30	−1.3269
−1.32431	13.9648	−0.00731	31	−1.32385
−1.32187	14.2088	−0.00731	32	−1.32141
−1.31943	14.4528	−0.0073	33	−1.31927
−1.31699	14.6968	−0.0073	34	−1.31714
−1.31454	14.9408	−0.00729	35	−1.31439
−1.3121	15.1848	−0.00729	36	−1.31226
−1.30966	15.4288	−0.00729	37	−1.30951
−1.30722	15.6728	−0.00728	38	−1.30707
−1.30478	15.9168	−0.00728	39	−1.30463
−1.30234	16.1608	−0.00727	40	−1.30188
−1.2999	16.4048	−0.00727	41	−1.29944
−1.29745	16.6488	−0.00726	42	−1.2973
−1.29501	16.8928	−0.00726	43	−1.29517
−1.29257	17.1368	−0.00725	44	−1.29211
−1.29013	17.3808	−0.00725	45	−1.28937
−1.28769	17.6248	−0.00725	46	−1.28723
−1.28525	17.8688	−0.00724	47	−1.28479
−1.28281	18.1128	−0.00724	48	−1.28235
−1.28036	18.3568	−0.00723	49	−1.28021
−1.27792	18.6008	−0.00723	50	−1.27747
−1.27548	18.8448	−0.00722	51	−1.27472
−1.27304	19.0888	−0.00722	52	−1.27289
−1.2706	19.3328	−0.00721	53	−1.27014
−1.26816	19.5768	−0.00721	54	−1.2677
−1.26572	19.8208	−0.0072	55	−1.26526
−1.26328	20.0648	−0.0072	56	−1.26343
−1.26083	20.3088	−0.00719	57	−1.26038
−1.25839	20.5528	−0.00719	58	−1.25793
−1.25595	20.7968	−0.00718	59	−1.25549
−1.25351	21.0408	−0.00718	60	−1.25366
−1.25107	21.2848	−0.00717	61	−1.25
−1.24863	21.5288	−0.00717	62	−1.24817
−1.24619	21.7728	−0.00716	63	−1.24542
−1.24374	22.0168	−0.00716	64	−1.24329
−1.2413	22.2608	−0.00716	65	−1.24115
−1.23886	22.5048	−0.00715	66	−1.23871
−1.23642	22.7488	−0.00715	67	−1.23566
−1.23398	22.9928	−0.00714	68	−1.23352
−1.23154	23.2368	−0.00714	69	−1.23108
−1.2291	23.4808	−0.00713	70	−1.22864
−1.22665	23.7248	−0.00713	71	−1.22681
−1.22421	23.9688	−0.00712	72	−1.22375
−1.22177	24.2128	−0.00712	73	−1.22131
−1.21933	24.4568	−0.00712	74	−1.21887
−1.21689	24.7008	−0.00711	75	−1.21674
−1.21445	24.9448	−0.00711	76	−1.21368
−1.21201	25.1888	−0.0071	77	−1.21155
−1.20956	25.4328	−0.0071	78	−1.2088
−1.20712	25.6768	−0.00709	79	−1.20667
−1.20468	25.9208	−0.00709	80	−1.20422
−1.20224	26.1648	−0.00708	81	−1.20209
−1.1998	26.4088	−0.00708	82	−1.19904
−1.19736	26.6528	−0.00708	83	−1.1972
−1.19492	26.8968	−0.00707	84	−1.19476
−1.19247	27.1408	−0.00707	85	−1.19202
−1.19003	27.3848	−0.00706	86	−1.18927
−1.18759	27.6288	−0.00706	87	−1.18713
−1.18515	27.8728	−0.00706	88	−1.185
−1.18271	28.1168	−0.00705	89	−1.18195
−1.18027	28.3608	−0.00705	90	−1.17981
−1.17783	28.6048	−0.00706	91	−1.17737
−1.17538	28.8488	−0.00706	92	−1.17493
−1.17294	29.0928	−0.00705	93	−1.17218
−1.1705	29.3368	−0.00705	94	−1.17035
−1.16806	29.5808	−0.00704	95	−1.16791
−1.16562	29.8248	−0.00704	96	−1.16547
−1.16318	30.0688	−0.00703	97	−1.16272
−1.16074	30.3128	−0.00703	98	−1.16089
−1.15829	30.5568	−0.00702	99	−1.15845
−1.15585	30.8008	−0.00702	100	−1.1554
−1.15341	31.0448	−0.00701	101	−1.15356
−1.15097	31.2888	−0.00701	102	−1.15112
−1.14853	31.5328	−0.00701	103	−1.14868
−1.14609	31.7768	−0.007	104	−1.14502
−1.14365	32.0208	−0.007	105	−1.14349
−1.1412	32.2648	−0.00699	106	−1.14075
−1.13876	32.5088	−0.00699	107	−1.13861
−1.13632	32.7528	−0.00698	108	−1.13647
−1.13388	32.9968	−0.00698	109	−1.13373
−1.13144	33.2408	−0.00697	110	−1.13098
−1.129	33.4848	−0.00697	111	−1.12885
−1.12656	33.7288	−0.00696	112	−1.1261
−1.12411	33.9728	−0.00696	113	−1.12396
−1.12167	34.2168	−0.00695	114	−1.12122
−1.11923	34.4608	−0.00695	115	−1.11877
−1.11679	34.7048	−0.00694	116	−1.11664
−1.11435	34.9488	−0.00694	117	−1.11389
−1.11191	35.1928	−0.00693	118	−1.11237
−1.10947	35.4368	−0.00693	119	−1.10901
−1.10703	35.6808	−0.00692	120	−1.10657
−1.10458	35.9248	−0.00692	121	−1.10382
−1.10214	36.1688	−0.00691	122	−1.10199
−1.0997	36.4128	−0.00691	123	−1.09894
−1.09726	36.6568	−0.0069	124	−1.09711
−1.09482	36.9008	−0.0069	125	−1.09436
−1.09238	37.1448	−0.00689	126	−1.09192
−1.08994	37.3888	−0.00689	127	−1.08978
−1.08749	37.6328	−0.00688	128	−1.08734
−1.08505	37.8768	−0.00688	129	−1.08521
−1.08261	38.1208	−0.00687	130	−1.08215
−1.08017	38.3648	−0.00687	131	−1.08032
−1.07773	38.6088	−0.00686	132	−1.07697
−1.07529	38.8528	−0.00686	133	−1.07483
−1.07285	39.0968	−0.00685	134	−1.07208
−1.0704	39.3408	−0.00684	135	−1.06995
−1.06796	39.5848	−0.00684	136	−1.0675
−1.06552	39.8288	−0.00683	137	−1.06567
−1.06308	40.0728	−0.00683	138	−1.06262
−1.06064	40.3168	−0.00682	139	−1.06079
−1.0582	40.5608	−0.00682	140	−1.05804
−1.05576	40.8048	−0.00681	141	−1.05591
−1.05331	41.0488	−0.00681	142	−1.05316
−1.05087	41.2928	−0.0068	143	−1.05042
−1.04843	41.5368	−0.00679	144	−1.04828
−1.04599	41.7808	−0.00679	145	−1.04523
−1.04355	42.0248	−0.00678	146	−1.0434
−1.04111	42.2688	−0.00678	147	−1.04126
−1.03867	42.5128	−0.00677	148	−1.03851
−1.03622	42.7568	−0.00677	149	−1.03638
−1.03378	43.0008	−0.00676	150	−1.03363
−1.03134	43.2448	−0.00676	151	−1.03058
−1.0289	43.4888	−0.00675	152	−1.02814
−1.02646	43.7328	−0.00675	153	−1.02631
−1.02402	43.9768	−0.00674	154	−1.02386
−1.02158	44.2208	−0.00673	155	−1.02081
−1.01913	44.4648	−0.00673	156	−1.01929
−1.01669	44.7088	−0.00672	157	−1.01654
−1.01425	44.9528	−0.00672	158	−1.01379
−1.01181	45.1968	−0.00671	159	−1.01166
−1.00937	45.4408	−0.0067	160	−1.00922
−1.00693	45.6848	−0.0067	161	−1.00647
−1.00449	45.9288	−0.00669	162	−1.00433
−1.00204	46.1728	−0.00668	163	−1.00159
−0.9996	46.4168	−0.00667	164	−0.99976
−0.99716	46.6608	−0.00667	165	−0.99731
−0.99472	46.9048	−0.00666	166	−0.99426
−0.99228	47.1488	−0.00666	167	−0.99213
−0.98984	47.3928	−0.00665	168	−0.9903
−0.9874	47.6368	−0.00665	169	−0.98755
−0.98496	47.8808	−0.00664	170	−0.9845
−0.98251	48.1248	−0.00664	171	−0.98267
−0.98007	48.3688	−0.00664	172	−0.98023
−0.97763	48.6128	−0.00664	173	−0.97748
−0.97519	48.8568	−0.00663	174	−0.97504
−0.97275	49.1008	−0.00663	175	−0.97229
−0.97031	49.3448	−0.00662	176	−0.96985
−0.96787	49.5888	−0.00661	177	−0.96771
−0.96542	49.8328	−0.00661	178	−0.96527
−0.96298	50.0768	−0.0066	179	−0.96314
−0.96054	50.3208	−0.00659	180	−0.96039
−0.9581	50.5648	−0.00659	181	−0.95825
−0.95566	50.8088	−0.00658	182	−0.95551
−0.95322	51.0528	−0.00657	183	−0.95245
−0.95078	51.2968	−0.00657	184	−0.95062
−0.94833	51.5408	−0.00656	185	−0.94849
−0.94589	51.7848	−0.00655	186	−0.94574
−0.94345	52.0288	−0.00655	187	−0.9433
−0.94101	52.2728	−0.00654	188	−0.94116
−0.93857	52.5168	−0.00654	189	−0.93872
−0.93613	52.7608	−0.00653	190	−0.93597
−0.93369	53.0048	−0.00652	191	−0.93292
−0.93124	53.2488	−0.00652	192	−0.93109
−0.9288	53.4928	−0.00651	193	−0.92835
−0.92636	53.7368	−0.0065	194	−0.9259
−0.92392	53.9808	−0.0065	195	−0.92407
−0.92148	54.2248	−0.00649	196	−0.92133
−0.91904	54.4688	−0.00648	197	−0.91888
−0.9166	54.7128	−0.00647	198	−0.91644
−0.91415	54.9568	−0.00647	199	−0.9137
−0.91171	55.2008	−0.00646	200	−0.91156
−0.90927	55.4448	−0.00645	201	−0.90881
−0.90683	55.6888	−0.00645	202	−0.90637
−0.90439	55.9328	−0.00644	203	−0.90393
−0.90195	56.1768	−0.00643	204	−0.9021
−0.89951	56.4208	−0.00642	205	−0.89966
−0.89706	56.6648	−0.00642	206	−0.89661
−0.89462	56.9088	−0.00641	207	−0.89447
−0.89218	57.1528	−0.0064	208	−0.89172
−0.88974	57.3968	−0.00639	209	−0.88928
−0.8873	57.6408	−0.00639	210	−0.88715
−0.88486	57.8848	−0.00638	211	−0.88471
−0.88242	58.1288	−0.00637	212	−0.88287
−0.87997	58.3728	−0.00636	213	−0.88013
−0.87753	58.6168	−0.00635	214	−0.87708
−0.87509	58.8608	−0.00635	215	−0.87463
−0.87265	59.1048	−0.00634	216	−0.87219
−0.87021	59.3488	−0.00633	217	−0.87006
−0.86777	59.5928	−0.00632	218	−0.86731
−0.86533	59.8368	−0.00631	219	−0.86517
−0.86289	60.0808	−0.00631	220	−0.86243
−0.86044	60.3248	−0.0063	221	−0.85999
−0.858	60.5688	−0.00629	222	−0.85754
−0.85556	60.8128	−0.00628	223	−0.85541
−0.85312	61.0568	−0.00627	224	−0.85266
−0.85068	61.3008	−0.00626	225	−0.85022
−0.84824	61.5448	−0.00626	226	−0.84808
−0.8458	61.7888	−0.00625	227	−0.84625
−0.84335	62.0328	−0.00624	228	−0.84351
−0.84091	62.2768	−0.00623	229	−0.84106
−0.83847	62.5208	−0.00622	230	−0.83832
−0.83603	62.7648	−0.00622	231	−0.83557
−0.83359	63.0088	−0.00621	232	−0.83344
−0.83115	63.2528	−0.0062	233	−0.83099
−0.82871	63.4968	−0.00619	234	−0.82886
−0.82626	63.7408	−0.00618	235	−0.82642
−0.82382	63.9848	−0.00617	236	−0.82336
−0.82138	64.2288	−0.00617	237	−0.82123
−0.81894	64.4728	−0.00616	238	−0.81848
−0.8165	64.7168	−0.00615	239	−0.81665
−0.81406	64.9608	−0.00614	240	−0.81451
−0.81162	65.2048	−0.00613	241	−0.81146
−0.80917	65.4488	−0.00612	242	−0.80872
−0.80673	65.6928	−0.00611	243	−0.80627
−0.80429	65.9368	−0.0061	244	−0.80444
−0.80185	66.1808	−0.00609	245	−0.8017
−0.79941	66.4248	−0.00608	246	−0.79895
−0.79697	66.6688	−0.00607	247	−0.79681
−0.79453	66.9128	−0.00606	248	−0.79407
−0.79208	67.1568	−0.00605	249	−0.79193
−0.78964	67.4008	−0.00604	250	−0.7898
−0.7872	67.6448	−0.00603	251	−0.78644
−0.78476	67.8888	−0.00602	252	−0.78491
−0.78232	68.1328	−0.00601	253	−0.78217
−0.77988	68.3768	−0.00599	254	−0.78003
−0.77744	68.6208	−0.00598	255	−0.77759
−0.77499	68.8648	−0.00597	256	−0.77515
−0.77255	69.1088	−0.00596	257	−0.7724
−0.77011	69.3528	−0.00595	258	−0.77026
−0.76767	69.5968	−0.00594	259	−0.76782
−0.76523	69.8408	−0.00593	260	−0.76538
−0.76279	70.0848	−0.00592	261	−0.76263
−0.76035	70.3288	−0.00591	262	−0.7608
−0.7579	70.5728	−0.00589	263	−0.75775
−0.75546	70.8168	−0.00588	264	−0.75562
−0.75302	71.0608	−0.00587	265	−0.75162
−0.75058	71.3048	−0.00586	266	−0.74927
−0.74814	71.5488	−0.00585	267	−0.74683
−0.7457	71.7928	−0.00583	268	−0.74439
−0.74326	72.0368	−0.00582	269	−0.74182
−0.74081	72.2808	−0.00581	270	−0.7395
−0.73837	72.5248	−0.0058	271	−0.737
−0.73593	72.7688	−0.00578	272	−0.73471
−0.73349	73.0128	−0.00577	273	−0.73224
−0.73105	73.2568	−0.00576	274	−0.72971
−0.72861	73.5008	−0.00575	275	−0.72726
−0.72617	73.7448	−0.00573	276	−0.72476
−0.72372	73.9888	−0.00572	277	−0.72238
−0.72128	74.2328	−0.00571	278	−0.71988
−0.71884	74.4768	−0.00569	279	−0.71759
−0.7164	74.7208	−0.00568	280	−0.71518
−0.71396	74.9648	−0.00566	281	−0.71274
−0.71152	75.2088	−0.00565	282	−0.71024
−0.70908	75.4528	−0.00564	283	−0.70776
−0.70664	75.6968	−0.00562	284	−0.70532
−0.70419	75.9408	−0.00561	285	−0.703
−0.70175	76.1848	−0.00559	286	−0.70044
−0.69931	76.4288	−0.00558	287	−0.69827
−0.69687	76.6728	−0.00556	288	−0.69574
−0.69443	76.9168	−0.00554	289	−0.69336
−0.69199	77.1608	−0.00553	290	−0.69058
−0.68955	77.4048	−0.00551	291	−0.68823
−0.6871	77.6488	−0.0055	292	−0.68573
−0.68466	77.8928	−0.00548	293	−0.68353
−0.68222	78.1368	−0.00546	294	−0.68088
−0.67978	78.3808	−0.00544	295	−0.67868
−0.67734	78.6248	−0.00542	296	−0.67621
−0.6749	78.8688	−0.00541	297	−0.67386
−0.67246	79.1128	−0.00539	298	−0.67142
−0.67001	79.3568	−0.00537	299	−0.66885
−0.66757	79.6008	−0.00535	300	−0.66638
−0.66513	79.8448	−0.00533	301	−0.66391
−0.66269	80.0888	−0.00531	302	−0.66162
−0.66025	80.3328	−0.00529	303	−0.65912
−0.65781	80.5768	−0.00527	304	−0.65698
−0.65537	80.8208	−0.00525	305	−0.65427
−0.65292	81.0648	−0.00523	306	−0.65189
−0.65048	81.3088	−0.00521	307	−0.64926
−0.64804	81.5528	−0.00518	308	−0.647
−0.6456	81.7968	−0.00516	309	−0.64444
−0.64316	82.0408	−0.00514	310	−0.64221
−0.64072	82.2848	−0.00511	311	−0.63959
−0.63828	82.5288	−0.00509	312	−0.63739
−0.63583	82.7728	−0.00506	313	−0.63467
−0.63339	83.0168	−0.00504	314	−0.63254
−0.63095	83.2608	−0.00502	315	−0.62988
−0.62851	83.5048	−0.00499	316	−0.62759
−0.62607	83.7488	−0.00496	317	−0.62485
−0.62363	83.9928	−0.00493	318	−0.62271
−0.62119	84.2368	−0.0049	319	−0.62018
−0.61874	84.4808	−0.00486	320	−0.61795
−0.6163	84.7248	−0.00483	321	−0.61527
−0.61386	84.9688	−0.00479	322	−0.61295
−0.61142	85.2128	−0.00475	323	−0.61026
−0.60898	85.4568	−0.00471	324	−0.60809
−0.60654	85.7008	−0.00466	325	−0.60547
−0.6041	85.9448	−0.00462	326	−0.60336
−0.60165	86.1888	−0.00457	327	−0.60083
−0.59921	86.4328	−0.00452	328	−0.59851
−0.59677	86.6768	−0.00447	329	−0.59601
−0.59433	86.9208	−0.00441	330	−0.59341
−0.59189	87.1648	−0.00435	331	−0.59094
−0.58945	87.4088	−0.00428	332	−0.58838
−0.58701	87.6528	−0.00421	333	−0.58618
−0.58456	87.8968	−0.00414	334	−0.58368
−0.58212	88.1408	−0.00406	335	−0.58148
−0.57968	88.3848	−0.00398	336	−0.57895
−0.57724	88.6288	−0.00389	337	−0.57651
−0.5748	88.8728	−0.00381	338	−0.57382
−0.57236	89.1168	−0.00372	339	−0.5715
−0.56992	89.3608	−0.00362	340	−0.56912
−0.56747	89.6048	−0.00353	341	−0.5669
−0.56503	89.8488	−0.00344	342	−0.56424
−0.56259	90.0928	−0.00335	343	−0.56201
−0.56015	90.3368	−0.00326	344	−0.55948
−0.55771	90.5808	−0.00317	345	−0.55716
−0.55527	90.8248	−0.00308	346	−0.55457
−0.55283	91.0688	−0.00299	347	−0.55234
−0.55039	91.3128	−0.0029	348	−0.54965
−0.54794	91.5568	−0.00282	349	−0.54733
−0.5455	91.8008	−0.00274	350	−0.54495
−0.54306	92.0448	−0.00266	351	−0.5426
−0.54062	92.2888	−0.00258	352	−0.54019
−0.53818	92.5328	−0.0025	353	−0.53781
−0.53574	92.7768	−0.00243	354	−0.53519
−0.5333	93.0208	−0.00235	355	−0.53272
−0.53085	93.2648	−0.00228	356	−0.53037
−0.52841	93.5088	−0.00221	357	−0.52792
−0.52597	93.7528	−0.00214	358	−0.5256
−0.52353	93.9968	−0.00208	359	−0.52313
−0.52109	94.2408	−0.00201	360	−0.52081
−0.51865	94.4848	−0.00195	361	−0.51828
−0.51621	94.7288	−0.00189	362	−0.51596
−0.51376	94.9728	−0.00183	363	−0.51328
−0.51132	95.2168	−0.00177	364	−0.5109
−0.50888	95.4608	−0.00172	365	−0.50842
−0.50644	95.7048	−0.00166	366	−0.50623
−0.504	95.9488	−0.00161	367	−0.50375
−0.50156	96.1928	−0.00156	368	−0.5014
−0.49912	96.4368	−0.00151	369	−0.49878
−0.49667	96.6808	−0.00146	370	−0.49646
−0.49423	96.9248	−0.00142	371	−0.49384
−0.49179	97.1688	−0.00137	372	−0.49155
−0.48935	97.4128	−0.00134	373	−0.48892
−0.48691	97.6568	−0.00129	374	−0.48654
−0.48447	97.9008	−0.00124	375	−0.48425
−0.48203	98.1448	−0.0012	376	−0.48184
−0.47958	98.3888	−0.00117	377	−0.47949
−0.47714	98.6328	−0.00113	378	−0.4769
−0.4747	98.8768	−0.00109	379	−0.47437
−0.47226	99.1208	−0.00106	380	−0.47192
−0.46982	99.3648	−0.00103	381	−0.46957
−0.46738	99.6088	−0.001	382	−0.46716
−0.46494	99.8528	−0.00096	383	−0.46494
−0.46249	100.097	−0.00094	384	−0.46237
−0.46005	100.341	−0.0009	385	−0.45993
−0.45761	100.585	−0.00088	386	−0.4574
−0.45517	100.829	−0.00085	387	−0.4549
−0.45273	101.073	−0.00082	388	−0.45236
−0.45029	101.317	−0.0008	389	−0.4502
−0.44785	101.561	−0.00077	390	−0.4476
−0.4454	101.805	−0.00075	391	−0.44534
−0.44296	102.049	−0.00073	392	−0.44266
−0.44052	102.293	−0.00071	393	−0.44049
−0.43808	102.537	−0.00069	394	−0.43784
−0.43564	102.781	−0.00067	395	−0.43552
−0.4332	103.025	−0.00065	396	−0.43289
−0.43076	103.269	−0.00063	397	−0.4307
−0.42831	103.513	−0.00061	398	−0.4281
−0.42587	103.757	−0.00059	399	−0.42584
−0.42343	104.001	−0.00057	400	−0.42337
−0.42099	104.245	−0.00055	401	−0.42108
−0.41855	104.489	−0.00053	402	−0.41852
−0.41611	104.733	−0.00051	403	−0.41586
−0.41367	104.977	−0.00049	404	−0.41339
−0.41122	105.221	−0.00048	405	−0.4108
−0.40878	105.465	−0.00046	406	−0.40866
−0.40634	105.709	−0.00044	407	−0.40616
−0.4039	105.953	−0.00042	408	−0.40387
−0.40146	106.197	−0.0004	409	−0.40125
−0.39902	106.441	−0.00039	410	−0.39899
−0.39658	106.685	−0.00037	411	−0.39633
−0.39414	106.929	−0.00035	412	−0.39398
−0.39169	107.173	−0.00033	413	−0.39139
−0.38925	107.417	−0.00031	414	−0.38913
−0.38681	107.661	−0.00029	415	−0.38672
−0.38437	107.905	−0.00027	416	−0.38434
−0.38193	108.149	−0.00025	417	−0.3819
−0.37949	108.393	−0.00023	418	−0.3793
−0.37705	108.637	−0.0002	419	−0.37692
−0.3746	108.881	−0.00018	420	−0.37436
−0.37216	109.125	−0.00016	421	−0.37198
−0.36972	109.369	−0.00013	422	−0.36945
−0.36728	109.613	−0.0001	423	−0.36728
−0.36484	109.857	−7.18E-05	424	−0.36462
−0.3624	110.101	−4.08E-05	425	−0.36234
−0.35996	110.345	−8.42E-06	426	−0.35974
−0.35751	110.589	2.73E-05	427	−0.35751
−0.35507	110.833	6.57E-05	428	−0.35495
−0.35263	111.077	0.000107	429	−0.35269
−0.35019	111.321	0.000152	430	−0.35022
−0.34775	111.565	0.000193	431	−0.34781
−0.34531	111.809	0.000234	432	−0.34561
−0.34287	112.053	0.000287	433	−0.34314
−0.34042	112.297	0.000346	434	−0.34088
−0.33798	112.541	0.000412	435	−0.33795
−0.33554	112.785	0.000488	436	−0.33557
−0.3331	113.029	0.00057	437	−0.33292
−0.33066	113.273	0.000658	438	−0.33066
−0.32822	113.517	0.000752	439	−0.32813
−0.32578	113.761	0.000851	440	−0.3259
−0.32333	114.005	0.000956	441	−0.3233
−0.32089	114.249	0.001065	442	−0.32111
−0.31845	114.493	0.00118	443	−0.31851
−0.31601	114.737	0.0013	444	−0.31632
−0.31357	114.981	0.001426	445	−0.31363
−0.31113	115.225	0.001559	446	−0.3111
−0.30869	115.469	0.001694	447	−0.30862
−0.30624	115.713	0.001833	448	−0.30624
−0.3038	115.957	0.001977	449	−0.30396
−0.30136	116.201	0.002124	450	−0.30167
−0.29892	116.445	0.002275	451	−0.29916
−0.29648	116.689	0.002431	452	−0.29654
−0.29404	116.933	0.00259	453	−0.29419
−0.2916	117.177	0.002752	454	−0.29178
−0.28915	117.421	0.002918	455	−0.2894
−0.28671	117.665	0.003087	456	−0.28708
−0.28427	117.909	0.003257	457	−0.28473
−0.28183	118.153	0.003426	458	−0.28226
−0.27939	118.397	0.003593	459	−0.27966
−0.27695	118.641	0.003755	460	−0.27728
−0.27451	118.885	0.003909	461	−0.27484
−0.27206	119.129	0.004051	462	−0.2724
−0.26962	119.373	0.00418	463	−0.27017
−0.26718	119.617	0.004295	464	−0.26761
−0.26474	119.861	0.004396	465	−0.26547
−0.2623	120.105	0.004487	466	−0.26285
−0.25986	120.349	0.004568	467	−0.26059
−0.25742	120.593	0.004641	468	−0.25781
−0.25497	120.837	0.004708	469	−0.25562
−0.25253	121.081	0.004769	470	−0.25308
−0.25009	121.325	0.004825	471	−0.25085
−0.24765	121.569	0.004878	472	−0.24814
−0.24521	121.813	0.004926	473	−0.24597
−0.24277	122.057	0.004972	474	−0.24365
−0.24033	122.301	0.005014	475	−0.24118
−0.23789	122.545	0.005055	476	−0.23868
−0.23544	122.789	0.005092	477	−0.23615
−0.233	123.033	0.005128	478	−0.23364
−0.23056	123.277	0.005163	479	−0.23129
−0.22812	123.521	0.005195	480	−0.22888
−0.22568	123.765	0.005226	481	−0.22638
−0.22324	124.009	0.005256	482	−0.22424
−0.2208	124.253	0.005284	483	−0.22162
−0.21835	124.497	0.005311	484	−0.21927
−0.21591	124.741	0.005338	485	−0.21652
−0.21347	124.985	0.005362	486	−0.21423
−0.21103	125.229	0.005386	487	−0.21164
−0.20859	125.473	0.005409	488	−0.20953
−0.20615	125.717	0.005432	489	−0.20691
−0.20371	125.961	0.005453	490	−0.20462
−0.20126	126.205	0.005473	491	−0.20203
−0.19882	126.449	0.005493	492	−0.19965
−0.19638	126.693	0.005512	493	−0.19708
−0.19394	126.937	0.005531	494	−0.19489
−0.1915	127.181	0.005549	495	−0.19214
−0.18906	127.425	0.005566	496	−0.19003
−0.18662	127.669	0.005583	497	−0.18729
−0.18417	127.913	0.005598	498	−0.18512
−0.18173	128.157	0.005614	499	−0.1825
−0.17929	128.401	0.005629	500	−0.18036
−0.17685	128.645	0.005643	501	−0.1777
−0.17441	128.889	0.005657	502	−0.17529
−0.17197	129.133	0.00567	503	−0.1727
−0.16953	129.377	0.005683	504	−0.17044
−0.16708	129.621	0.005695	505	−0.16782
−0.16464	129.865	0.005706	506	−0.16541
−0.1622	130.109	0.005717	507	−0.16315
−0.15976	130.353	0.005727	508	−0.16086
−0.15732	130.597	0.005737	509	−0.15817
−0.15488	130.841	0.005746	510	−0.15567
−0.15244	131.085	0.005754	511	−0.1532
−0.14999	131.329	0.005762	512	−0.1507
−0.14755	131.573	0.005769	513	−0.14835
−0.14511	131.817	0.005775	514	−0.14603
−0.14267	132.061	0.005781	515	−0.14374
−0.14023	132.305	0.005786	516	−0.14114
−0.13779	132.549	0.005791	517	−0.13886
−0.13535	132.793	0.005795	518	−0.13614
−0.1329	133.037	0.005798	519	−0.13391
−0.13046	133.281	0.0058	520	−0.13123
−0.12802	133.525	0.005802	521	−0.12891
−0.12558	133.769	0.005803	522	−0.1264
−0.12314	134.013	0.005804	523	−0.12427
−0.1207	134.257	0.005803	524	−0.12158
−0.11826	134.501	0.005802	525	−0.1192
−0.11581	134.745	0.0058	526	−0.1167
−0.11337	134.989	0.005797	527	−0.11435
−0.11093	135.233	0.005793	528	−0.11194
−0.10849	135.477	0.005789	529	−0.10938
−0.10605	135.721	0.005783	530	−0.10675
−0.10361	135.965	0.005777	531	−0.10452
−0.10117	136.209	0.005769	532	−0.10208
−0.09872	136.453	0.005761	533	−0.09982
−0.09628	136.697	0.005751	534	−0.09729
−0.09384	136.941	0.005739	535	−0.09473
−0.0914	137.185	0.005736	536	−0.09222
−0.08896	137.429	0.005721	537	−0.08975
−0.08652	137.673	0.005703	538	−0.08725
−0.08408	137.917	0.005684	539	−0.08487
−0.08163	138.161	0.005662	540	−0.08252
−0.07919	138.405	0.005638	541	−0.08023
−0.07675	138.649	0.005611	542	−0.07767
−0.07431	138.893	0.005581	543	−0.0752
−0.07187	139.137	0.005549	544	−0.07257
−0.06943	139.381	0.005513	545	−0.07019
−0.06699	139.625	0.005475	546	−0.06769
−0.06454	139.869	0.005435	547	−0.0654
−0.0621	140.113	0.005393	548	−0.06308
−0.05966	140.357	0.005352	549	−0.06058
−0.05722	140.601	0.00531	550	−0.05814
−0.05478	140.845	0.005267	551	−0.05548
−0.05234	141.089	0.005221	552	−0.05307
−0.0499	141.333	0.00517	553	−0.05054
−0.04745	141.577	0.005114	554	−0.04813
−0.04501	141.821	0.005051	555	−0.0449
−0.04257	142.065	0.004995	556	−0.04241
−0.04013	142.309	0.004895	557	−0.04015
−0.03769	142.553	0.004778	558	−0.03764
−0.03525	142.797	0.004626	559	−0.03503
−0.03281	143.041	0.004415	560	−0.03243
−0.03037	143.285	0.004101	561	−0.03004
−0.02792	143.529	0.003648	562	−0.0277
−0.02548	143.773	0.003162	563	−0.02532
−0.02304	144.017	0.002784	564	−0.02288
−0.0206	144.261	0.002517	565	−0.02041
−0.01816	144.505	0.002321	566	−0.01797
−0.01572	144.749	0.002171	567	−0.01531
−0.01328	144.993	0.002053	568	−0.01286
−0.01083	145.237	0.00196	569	−0.01051
−0.00839	145.481	0.001885	570	−0.00816
−0.00595	145.725	0.001815	571	−0.00572
−0.00351	145.969	0.001759	572	−0.00338
−0.00107	146.213	0.001713	573	−0.00081
0.001373	146.457	0.001676	574	0.00159

**Scheme 1:**
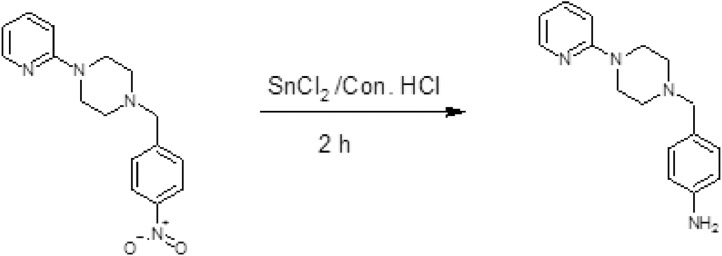
Synthesis of PPMA.

**Table 2:** MS rate of corrosion on CO_2_ medium at room temperature.

ba	bc	Ecorr,	E corr.,	j corr.	Icorr.	Corrosion	Polarization	E Begin	E End
(V/dec)	(V/dec)	Calc (V)	Obs (V)	(A/cm²)	(A)	rate (mm/year)	resistance (Ω)	(V)	(V)
0.17394	0.045221	−0.35222	−0.35938	0.000221	0.000221	2.5625	70.681	−0.4454	−0.32333

## 2 Experimental Design, Materials and Methods

### 2.1 Materials

1-(pyridin-2-yl)piperazine, 98.0%; 1-[(4-nitrophenyl)methyl]-4-(pyridin-2-yl)piperazine, 98%; 4-nitrobenzaldehyde, 98.5%; 1,2-dichloroethane were bought from AAPPTEC, USA. Sodiumtriacetoxy borohydride, 99.0%; and other reagents and solvents were purchased from HiMedia Laboratories Pvt. Ltd. (Mumbai, India). Every one of the chemicals was used without auxiliary purification. The entire aqueous solutions to be prepared by nanopure water. All equipment and glassware's are washed through acetone, rinsed by deionized water (DIW) and dehydrated with air searing owen at 100 °C, then it was used throughout the studies.

### 2.2 Synthesis of 4-{[4-(pyridin-2-yl)piperazin-1-yl]methyl}aniline (PPMA)

**Fig. 3:**
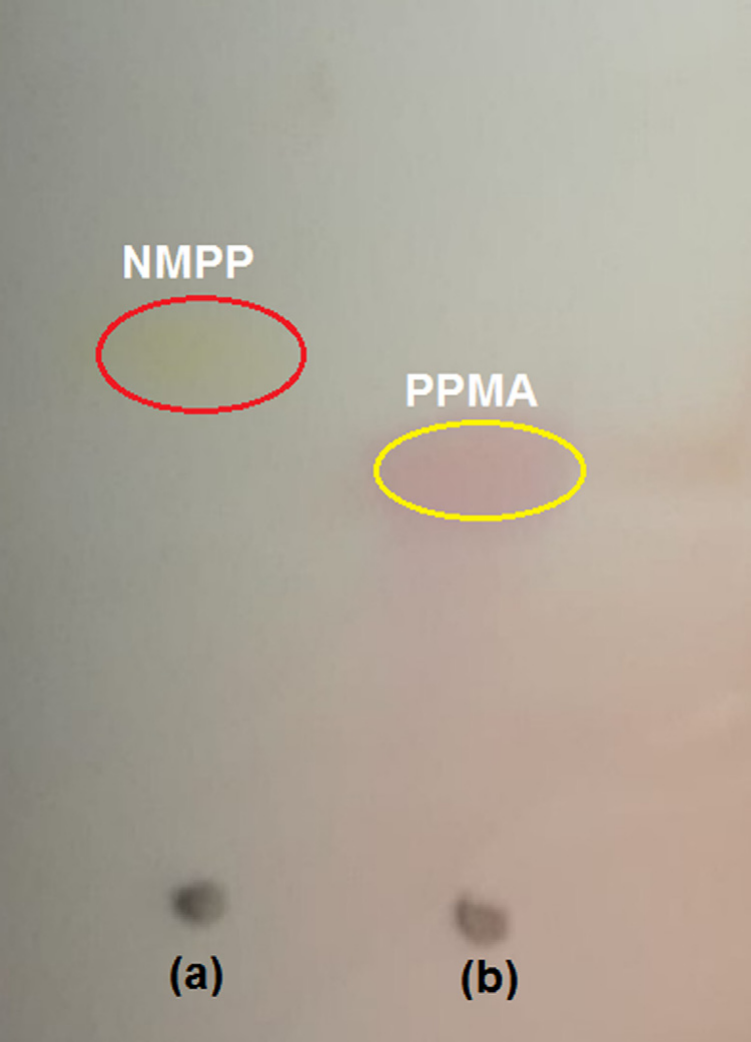
TLC of (a) Parent NMPP and (b) PPMA.

The solution of 1-[(4-nitrophenyl)methyl]-4-(pyridin-2-yl)piperazine (NMPP) Scheme S1 (6 g, 0.020 mol) reported in 50 mL 12N HCl, SnCl_2_ .2H_2_O (18.1 g, 0.080 mol) was added portion wise at RT [2], [7]. The resulting reaction mass was stirred at RT for 2 h, See Scheme 1. The progress of the reaction was monitored by TLC in Fig. 3. The reaction mixture was diluted with 250 mL of cold water. The solution was basified to pH 9–10 with 40% NaOH and the aqueous layer was extracted with ethyl acetate (3 × 250 mL), washed with water (2 × 250 mL), brine (1 × 300 mL) and dried over anhydrous Na_2_SO_4_.The solvent was concentrated under reduced pressure to give the titled compound. Light brown solid; 4.73 g. In this product is used as a catalyst for corrosion inhibition for mild steel at elevated temperature in CO_2_ medium.

### 2.3 Characterization techniques

The high resolution scanning electron microscopy (HR-SEM) was carried out on a FEI Quanta FEG 200 instrument facility at 25 °C. The taster was equipped by introduce a minute amount of primed material on a carbon coated copper network and allowing the solvent to evaporate. FT-IR spectra were recorded with thermo scientific spectrometer, model no. iS5 equipped with attenuated total reflectance (ATR) competence which is implemented by Zn-Se crystal detector. Each spectrum was recorded with an achievement time of 18 s. The FT-IR dimension was scanned at a range from 4000 to 400 cm^–1^.

Interpretation of FT-IR and NMR spectral data of PPMA is mainly focused on the MS interaction with PPMA vibrations at 408.0 cm^−1^ indicate that C-N-O bending, and 3150.4, 3270.0 cm^−1^ represent the NH and NH_2_ stretching vibrations is confirmed by the PPMA adsorbed on the MS surfaces (see Supporting Information Table S1).

## Ethics Statement

This article conforms to Elsevier's standards of ethical publishing.

## Data Availability

The data is available with this article.

## CRediT authorship contribution statement

**Raman Govindhan:** Conceptualization, Writing – original draft, Supervision. **Srinivasan Anbalagan:** Conceptualization, Data curation, Methodology. **Meenakshisundaram Ravishankar:** Writing – review & editing.

## Declaration of Competing Interest

The authors declare that they have no known competing financial Interests or personal relationships which have or could be perceived to have influenced the work reported in this article.

## Appendix Supplementary Materials

Image, application 1

Image, application 2

## Footnotes

Supplementary material associated with this article can be found in the online version at doi:10.1016/j.dib.2021.107492.
