# Supplementary material for: HR-SEM and FT-IR dataset for green corrosion inhibition activity of 4-{[4-(pyridin-2-yl)piperazin-1-yl]methyl}aniline at CO2 atmosphere
Source: Data Brief. 2021 Oct 18;39:107492. doi: 10.1016/j.dib.2021.107492 (PMC8554451; doi:10.1016/j.dib.2021.107492)
Supplement: Supplementary file 1 [file mmc1.doc]

**Supplementary Material**

**HR-SEM and FT-IR dataset for Green corrosion inhibition activity of 4-{[4-(pyridin-2-yl)piperazin-1-yl]methyl}aniline at CO_2_ atmosphere**

Raman Govindhan^a*^, Srinivasan Anbalagan^a^, and Meenakshisundaram Ravishankar^b^

^a^  Department of Chemistry, Sri Moogambigai Arts & Science College (women), affiliated to University, Dharmapuri 636 805, Tamilnadu, India.

^b^  Department of Chemistry, Laxminarayana College of Arts & Science (women), affiliated to University, Dharmapuri 636 705, Tamilnadu, India.

^c^ Department of Chemistry, Rajah Serfoji Government College (Autonomus), Thanjavur 613 005, Tamilnadu, India.


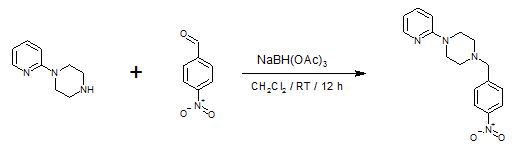


# Scheme S1

**Scheme S1. Synthesis of 1-[(4-nitrophenyl)methyl]-4-(pyridin-2-yl)piperazine (NMPP)**

A dry 250 mL round bottom flask with magnetic stirring bar was charged consecutively with 1-(pyridin-2-yl)piperazine (5 g, 0.036 mol), 4-nitrobenzaldehyde (5.1 g, 0.033 mol) and 1,2-dichloroethane (100 mL) in nitrogen atmosphere then treated with sodiumtriacetoxy borohydride (9.7 g, 0.046 mol). The resulting reaction mixture was stirred at RT under a Nitrogen atmosphere for 12 hr. The progress of the reaction was monitored by TLC in **Fig. 3** [**2**]. The reaction mass was quenched by adding aqueous saturated NaHCO_3_ (50 mL), and extracted with ethyl acetate (2x150 mL). The combined organic layer was washed with water (1x200 mL), saturated NaCl (1x300 mL), dried over Na_2_SO_4_ and the solvents were removed under reduced pressure. Pale yellow solid, 7.38 g.
